# Predicting Dengue Fever Outbreaks in French Guiana Using Climate Indicators

**DOI:** 10.1371/journal.pntd.0004681

**Published:** 2016-04-29

**Authors:** Antoine Adde, Pascal Roucou, Morgan Mangeas, Vanessa Ardillon, Jean-Claude Desenclos, Dominique Rousset, Romain Girod, Sébastien Briolant, Philippe Quenel, Claude Flamand

**Affiliations:** 1 Unité d’épidémiologie, Institut Pasteur de la Guyane, Cayenne, Guyane; 2 Unité d’entomologie médicale, Institut Pasteur de la Guyane, Cayenne, Guyane; 3 Centre de Recherches de Climatologie, UMR6282 Biogéosciences, CNRS Université de Bourgogne Franche-Comté, Dijon, France; 4 Maison de la Télédétection, UMR 228 ESPACE-DEV, Institut de Recherche pour le Développement, Montpellier, France; 5 Cellule de l’Institut de Veille Sanitaire en Régions Antilles - Guyane, Cayenne, Guyane; 6 Institut de Veille Sanitaire, Saint-Maurice, France; 7 Unité de virologie, Institut Pasteur de la Guyane, Cayenne, Guyane; 8 Direction Interarmées du Service de Santé en Guyane, Cayenne, Guyane; 9 Institut de Recherche Biomédicale des Armées, Brétigny sur Orge, France; 10 Laboratoire d’Etudes et de Recherche en Santé-Environnement, Ecole des Hautes Etudes en Santé Publique, Rennes, France; Santa Fe Institute, UNITED STATES

## Abstract

**Background:**

Dengue fever epidemic dynamics are driven by complex interactions between hosts, vectors and viruses. Associations between climate and dengue have been studied around the world, but the results have shown that the impact of the climate can vary widely from one study site to another. In French Guiana, climate-based models are not available to assist in developing an early warning system. This study aims to evaluate the potential of using oceanic and atmospheric conditions to help predict dengue fever outbreaks in French Guiana.

**Methodology/Principal Findings:**

Lagged correlations and composite analyses were performed to identify the climatic conditions that characterized a typical epidemic year and to define the best indices for predicting dengue fever outbreaks during the period 1991–2013. A logistic regression was then performed to build a forecast model. We demonstrate that a model based on summer Equatorial Pacific Ocean sea surface temperatures and Azores High sea-level pressure had predictive value and was able to predict 80% of the outbreaks while incorrectly predicting only 15% of the non-epidemic years. Predictions for 2014–2015 were consistent with the observed non-epidemic conditions, and an outbreak in early 2016 was predicted.

**Conclusions/Significance:**

These findings indicate that outbreak resurgence can be modeled using a simple combination of climate indicators. This might be useful for anticipating public health actions to mitigate the effects of major outbreaks, particularly in areas where resources are limited and medical infrastructures are generally insufficient.

## Author Summary

Climatic determinants are amongst the most frequently cited in studies aimed at understanding and explaining the dynamics of vector-borne infections, and dengue in particular. French Guiana, a French overseas territory in which the vector *Aedes aegypti* is well established, experiences an epidemic cycle of dengue with large and prolonged epidemics occurring approximately every 3 years. Dengue is one of the most prioritized infectious diseases, and it requires an intense mobilization of local public health authorities, health services, and health professional and vector control services. A specific surveillance, preparedness and response plan has been developed based upon these needs. Gaining an accurate understanding of the drivers of dengue transmission is required to develop a model to predict the risk of an epidemic and to plan activities aimed at controlling it. Here, we assessed the effects of climatic factors on dengue spread to develop a predictive model of the epidemics in French Guiana on a country-wide scale. The goal of the model is to anticipate and plan both preventive and control activities. Given climate conditions, the model predicts that a dengue epidemic is likely to occur in early 2016. These conditions, which are favorable for *Aedes* mosquito proliferation, could also enhance the diffusion of other arboviruses, such as the Zika virus, in northeastern South America.

## Introduction

Dengue fever (DF) is one of the most important mosquito-borne diseases in the world [1,2]. Recent estimates indicate that there are 390 million dengue infections per year, of which 96 million manifest as disease [3]. Infection is caused by the dengue virus (DENV), which has four closely related serotypes (DENV1 to DENV4) [4] that are transmitted to humans by infected *Aedes sp*. mosquitos. Infection produces a spectrum of illnesses that range from indiscernible or mildly nonspecific febrile syndrome to severe disease forms, including dengue hemorrhagic fever (DHF) and dengue shock syndrome (DSS). Currently, there are no specific dengue therapeutics, and prevention strategies are limited to vector control measures [5]. The recent development of the first dengue vaccine represents a major advance in our ability to control the disease [6–8].

In Latin American and Caribbean countries, the reintroduction and dissemination of *Aedes aegypti* occurred in the 1970s [9]. Since then, regular outbreaks have occurred on a 3- to 5-year cycle, and an increase has been observed in the frequency of severe forms of dengue [10]. In French Guiana, a French overseas territory of 250,000 inhabitants that is located in South America along the Atlantic Ocean, the epidemiology of dengue evolved from endemo-epidemic to hyper-endemic conditions over the two last decades [11]. Since the first DHF cases were reported in 1992 [12], transmission in French Guiana has followed a seasonal pattern that is punctuated every few years by major outbreaks that have been linked to the circulation of one or two predominant serotypes [11,13].

With the increasing frequency of such epidemics and the associated public health and socioeconomic issues [14], the surveillance, prevention and control of dengue have become social, political and public health challenges that require specific preparedness activities, particularly in areas where resources are limited. Although dengue ecology is known to be influenced by a complex multi-scale interplay of intrinsic factors that include human host demographics, vectors, and viruses and extrinsic factors that include environmental, meteorological and climate conditions, the factors that drive DF epidemics are not yet clearly understood [15–22].

Interactions between climate and DF outbreaks have been studied worldwide [23–34]. The findings of these studies suggest that the effects of climate parameters on the incidence of DF can vary widely from one study site to another [35,36] and that they depend largely on local context and epidemiological patterns. In South America, studies designed to determine the impact of climate on DF epidemics have suggested a role for El Niño events as triggers for epidemics [35]. El Niño conditions are likely to influence DF dynamics indirectly by modulating temperature, humidity and rainfall. In French Guiana, the sole study that focused on the DF-climate relationship identified a synchronous positive association between the occurrence of El Niño events, warmer temperatures, less abundant rainfall and dengue epidemics [37]. These results were obtained using basic analytical methods, and the study investigated El Niño conditions on a coarse annual scale. These results must be explored further, particularly if they are to be useful for prediction purposes. Moreover, the quality of epidemiological data (i.e., estimated suspected cases) that were available for the period covered by the study (1963–1993) was highly questionable.

Thus, even if associations between El Niño conditions, meteorology and DF epidemics are suspected, there is currently no climate-based model to assist in developing an early warning system in French Guiana. Based on the *a priori* hypothesis put forward in Gagnon et al. [37], the current study explores the potential of integrating sea surface temperature (SST) conditions to serve as a proxy for epidemic risk several months before the onset of a DF outbreak. In addition, we push our analysis further by also investigating the use of large-scale atmospheric circulation and regional climate patterns as more optimal indicators for predicting outbreaks. Using a long-term epidemiological surveillance dataset, this study explores the possibility of using a predictive model to assist public health authorities in implementing timely, appropriate and efficient prevention and mitigation strategies.

## Methods

### Study area

French Guiana is an overseas region of France that is located in northern South America between Brazil and Surinam. The climate is equatorial, hot and wet. The monthly mean temperatures (near 27°C) and relative humidity, which rarely falls below 80%, are nearly constant year-round. Spatial variations across the territory, particularly in the coastal area (regrouping 90% of the population), are low. Among meteorological parameters, only rainfall presents significant seasonal variations influenced by the migration of the intertropical convergence zone (ITCZ). The mean annual cumulative rainfall is approximately three meters, and there are four alternating seasons: a long rainy season from the beginning of April to mid-July, a long dry season from mid-July to mid-November, a short rainy season from mid-November to mid-February, and a shorter dry season from mid-February to the beginning of April. Large inter-annual variations in the total cumulated rainfall have been observed, and they are partly governed by large-scale atmospheric and oceanic patterns. A well-documented issue is the impact of El Niño conditions. During El Niño (La Niña) years, a rainfall deficit (surplus) occurs in French Guiana [38–42].

### Epidemiological data

Epidemiologic data on DF were obtained from two different sources, depending on the collection period. For the data from 1991–2006, a surveillance system was used, and data were based on a weekly census of biologically confirmed cases (BCCs) that were recorded by the Arbovirus National Reference Centre, which is based at the Pasteur Institute of French Guiana. In 2006, a multi-source surveillance system was implemented by the Regional Epidemiology Unit of the Institut de Veille Sanitaire that included all seven biological laboratories (public hospital and private laboratories) that are located in the coastal area. Concurrently, in 2006, a new dengue diagnostic test based on NS1 antigen detection was made available to all laboratories, and it contributed substantially to improving surveillance. Cases were biologically confirmed by isolating the virus and detecting viral RNA using reverse-transcription PCR (RT-PCR), NS1 antigen detection methods or serological tests that are based on an immunoglobulin M (IgM)-capture enzyme-linked immunosorbent assay (MAC-ELISA) [13]. This surveillance system was authorized by the French Data Protection Agency (CNIL, N°1213498). The DF incidence rates in French Guiana, which are defined as the yearly number of cases/100 000 inhabitants, were calculated for the 1991–2013 period. A standardization procedure was performed separately for the 1991 –April 2006 and May 2006–2013 periods. For this procedure, we used a z-score scaling method to take into account the improvement in the epidemiological surveillance system that was observed in April 2006. This approach led to a trend toward increasing numbers of cases and enabled us to work with a single dataset. The standardization was calculated using the following equation:

$$z=\frac{x-\overset{´}{x}}{\sigma }$$

where x, $\overset{´}{x}$ and *σ* were the observed value, mean and standard deviation of the incidence, respectively. The epidemic years were identified by applying the tercile method to the normalized and standardized sum of the monthly cases that occurred during the high incidence period. The first tercile was defined as the “low” incidence group, the second was defined as the “intermediate” incidence group, and the third was defined as the “high” incidence group.

### Climate data

We used sets of meteorological parameters and large-scale atmospheric and global SST data for 1990–2013 for this study. Meteorological records included rainfall, temperature and relative humidity and were obtained from Météo-France. We calculated monthly country means from these daily station data throughout the study period. A set of atmospheric and SST predictors was constructed from the ERA-Interim reanalysis data that were obtained from the European Centre for Medium-Range Weather Forecasts [43]. The ERA-Interim system assimilates observations and outputs using a forecast model. The climate fields were available at a 0.75°x0.75° spatial resolution and 60 vertical levels.

### Identifying the potential predictive climate parameters

First, time-lagged Spearman’s correlations were used to explore associations among the occurrence of El Niño events, warmer temperatures, less abundant rainfall and dengue epidemics as previously suggested by Gagnon et al. [37]. Different El Niño-related SST and sea level pressure (SLP) indices were tested, including Niño areas 1 to 4, the Southern Oscillation Index (SOI) and the multivariate ENSO index (MEI). Yearly DF incidences were correlated with the monthly climate data for each month in the preceding year.

Second, the relationships between DF outbreaks and large-scale atmospheric and oceanic parameters were assessed using a composite analysis [44] and following an exploratory approach. The composite method was used to identify the conditions that characterized a typical epidemic year and to assess the optimal indices to use to analyze DF outbreak predictions. Two samples (the composites) were built that contained the climate data for both epidemic and non-epidemic years. The differences between the means of the two samples were calculated at each grid point between 50°N-50°S and 150°W-0°E. The significance of the differences between the epidemic and non-epidemic years was assessed using Student’s *t*-tests.

### Building a climate-based forecasting model

Considering that major outbreaks affect a very large part of French Guiana, we built a climate-based forecast model using the climate factors identified as having an influence on DF at a country level. A logistic binomial (epidemic or non-epidemic year) regression model was used. If *p* is the probability of an outbreak, then $\left(\frac{p}{1-p}\right)$ is the odds of observing an outbreak. Thus, the following logistic regression model was used:

$$log\left(\frac{p}{1-p}\right)=\beta_{0}+\overset{k}{\underset{i=1}{\sum^{\;}}}\beta_{i}x_{i}$$

where *log* represents the natural logarithm, *k* represents the number of selected climate predictors, *β*_*i*_ represents the coefficient of the *i*^th^ predictor and *x*_*i*_ represents the *i*^th^ predictor. This model can be restated as follows:

$$p=\frac{\text{exp}(\beta_{0}+{\sum^{\;}}_{i=1}^{k}\beta_{i}x_{i})}{1+\text{exp}\left(\beta_{0}+{\sum^{\;}}_{i=1}^{k}\beta_{i}x_{i}\right)}$$

Logistic binomial regressions were fitted using univariate and multivariate methods by applying all of the possible predictor combinations. The model that maximized the AUC (area under the curve) from the receiver operating characteristic (ROC) analysis [45] and minimized the AIC (Akaike information criterion) [46] was selected. The final model performances were evaluated by calculating ROC scores and cross-validating the data [47]. The ROC is a method of testing the skill of categorical forecasts using the hit rate (HR) and false alarm rate (FAR). The HR indicates the proportion of epidemic years that were categorically forecast (sensitivity). It ranges from 0 to 1 (1 being desirable) and is calculated as follows:

$$HR=\frac{Hits}{Hits+Misses}$$

The FAR is the proportion of non-epidemic years that were forecast as epidemic years (1-specificity). It ranges from 0 to 1 (0 being desirable) and is defined as follows:

$$FAR=\frac{False\;alarms}{Hits+False\;alarms}$$

Second, a cross-validation on chunks of multiple years was performed to measure the model stability. Leave-one-out cross-validation (LOOCV) (i.e. 23-fold) was used. The model was refitted according to the number of observations, and the observations were then temporarily removed one by one. The resulting LOOCV δ was the cross-validation estimate of prediction error.

## Results

### Dengue fever multiannual seasonality and year-to-year variability

**Fig 1:**
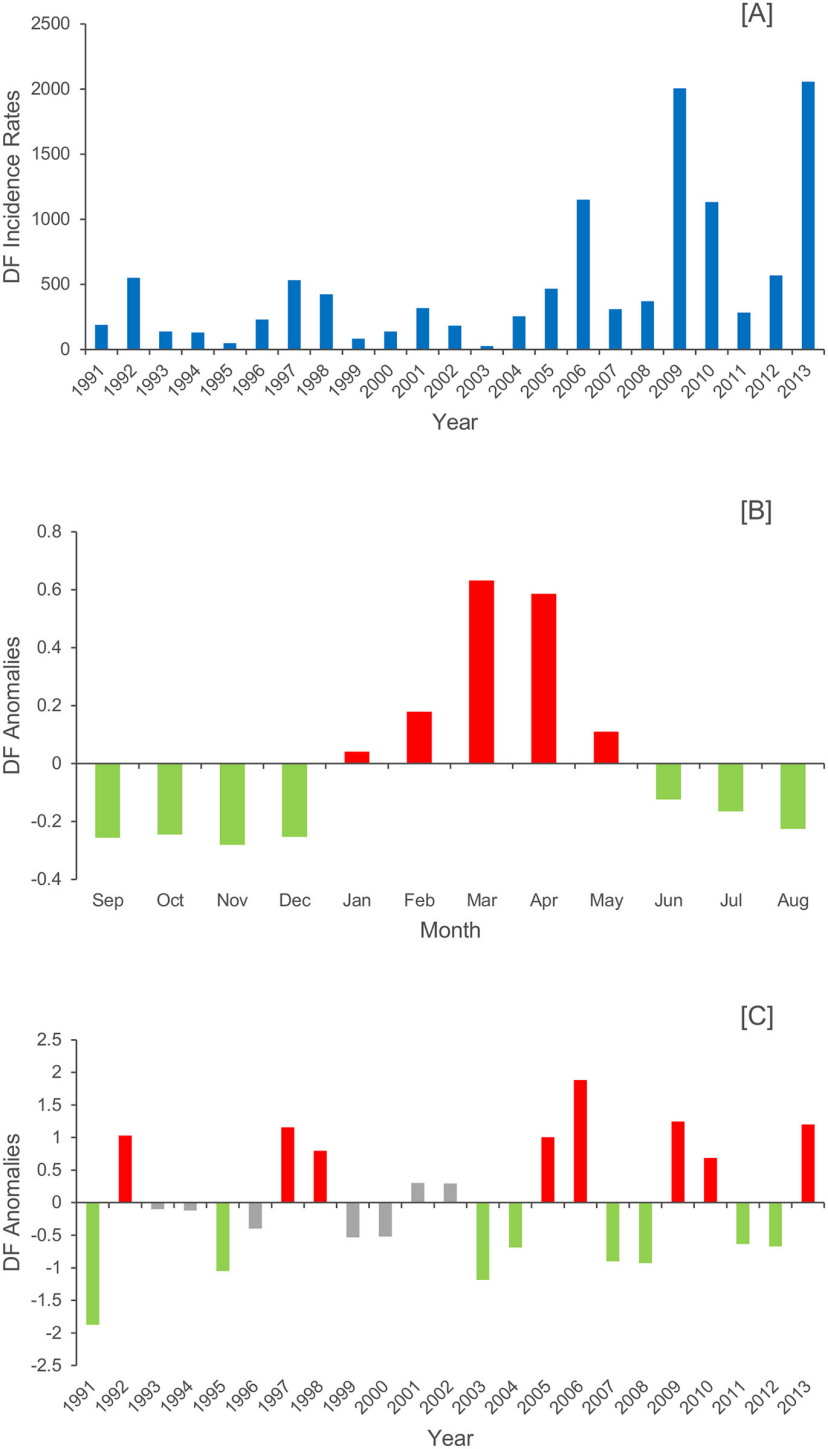
Dengue fever (DF) dynamics (1991–2013) in French Guiana. (*A*) DF annual incidence rates per 100 000 inhabitants. (*B*) Monthly mean DF incidence rate standardized anomalies. (*C*) Normalized and standardized DF annual incidence rates during the high incidence period (DF-HIR).

The year-to-year variability in DF incidence rates in French Guiana was described over a 23-year period from 1991–2013 (Fig 1A). The monthly mean cycle of DF standardized anomalies showed that there was strong seasonality (Fig 1B). The mean onset of the high incidence period was in January (positive anomalies) during the short rainy season. DF case peaks generally occurred in March, and the anomalies then decreased until May (negative in June). The high incidence period was therefore defined as January–May. Eight major outbreaks (third tercile) were identified: 1992, 1997, 1998, 2005, 2006, 2009, 2010 and 2013 (Fig 1C).

### Primary assessment of the climatic impact on DF

**Fig 2:**
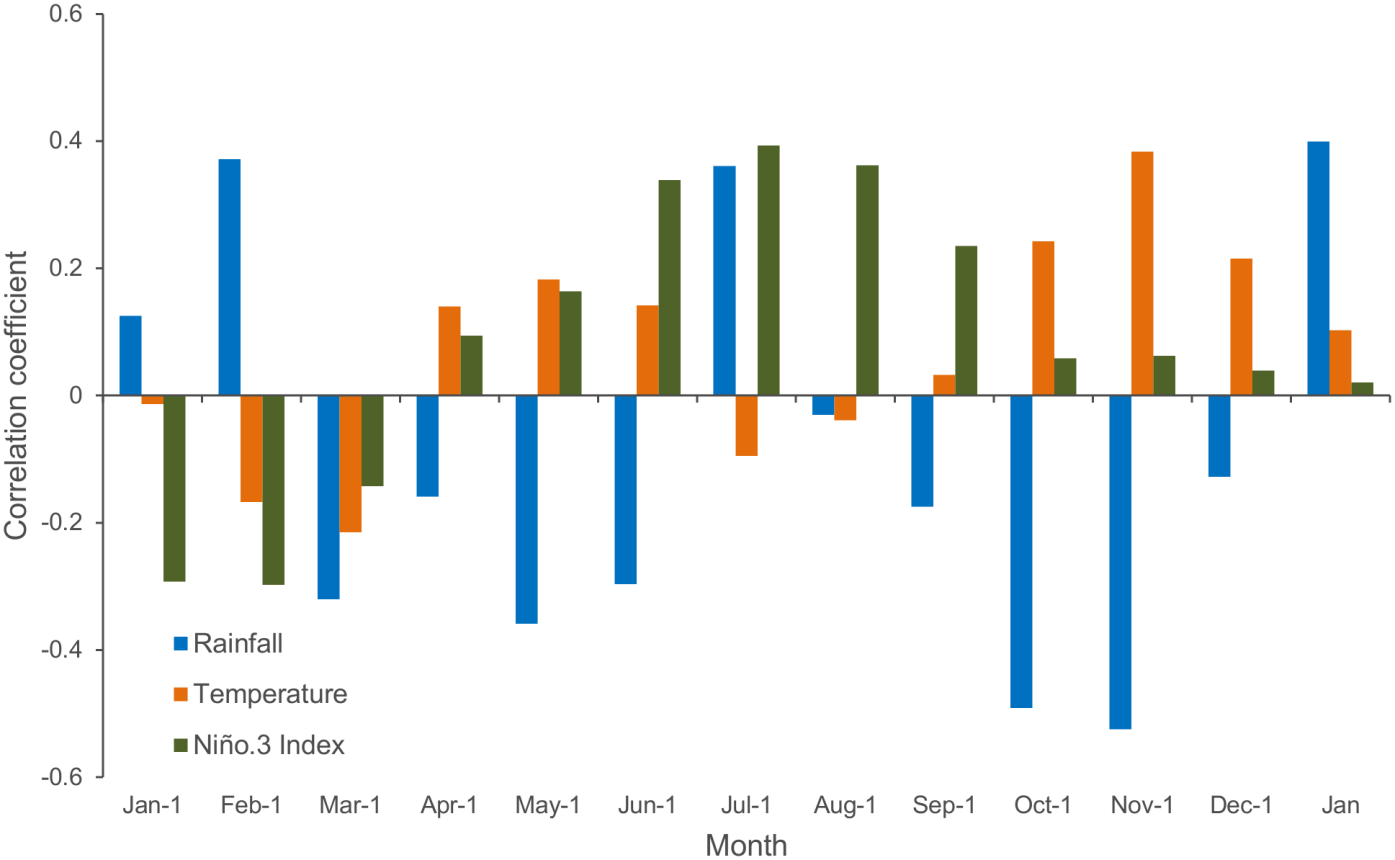
Spearman’s lagged correlation between dengue fever annual incidence rates and monthly climate parameters. Red stars: significant values at the 95% confidence interval.

Spearman’s lagged correlations indicated the presence of associations between DF and monthly pre-epidemic climate factors (Fig 2). Among El Niño indicators, the Niño 3 area index showed the highest correspondence with DF. A significant negative correlation was observed between DF and rainfall in October (r = -0.49, p-value = 0.02) and November (r = -0.52, p-value = 0.01), which are one and three months before the mean onset of the epidemics, respectively. El Niño event-related indices and temperatures were not significantly associated with DF (p-value > 0.05). However, an interesting, persistent, positive and nearly significant correlation was observed between the Niño 3 area index and DF during the summer months, and this relationship deserves further investigation.

**Fig 3:**
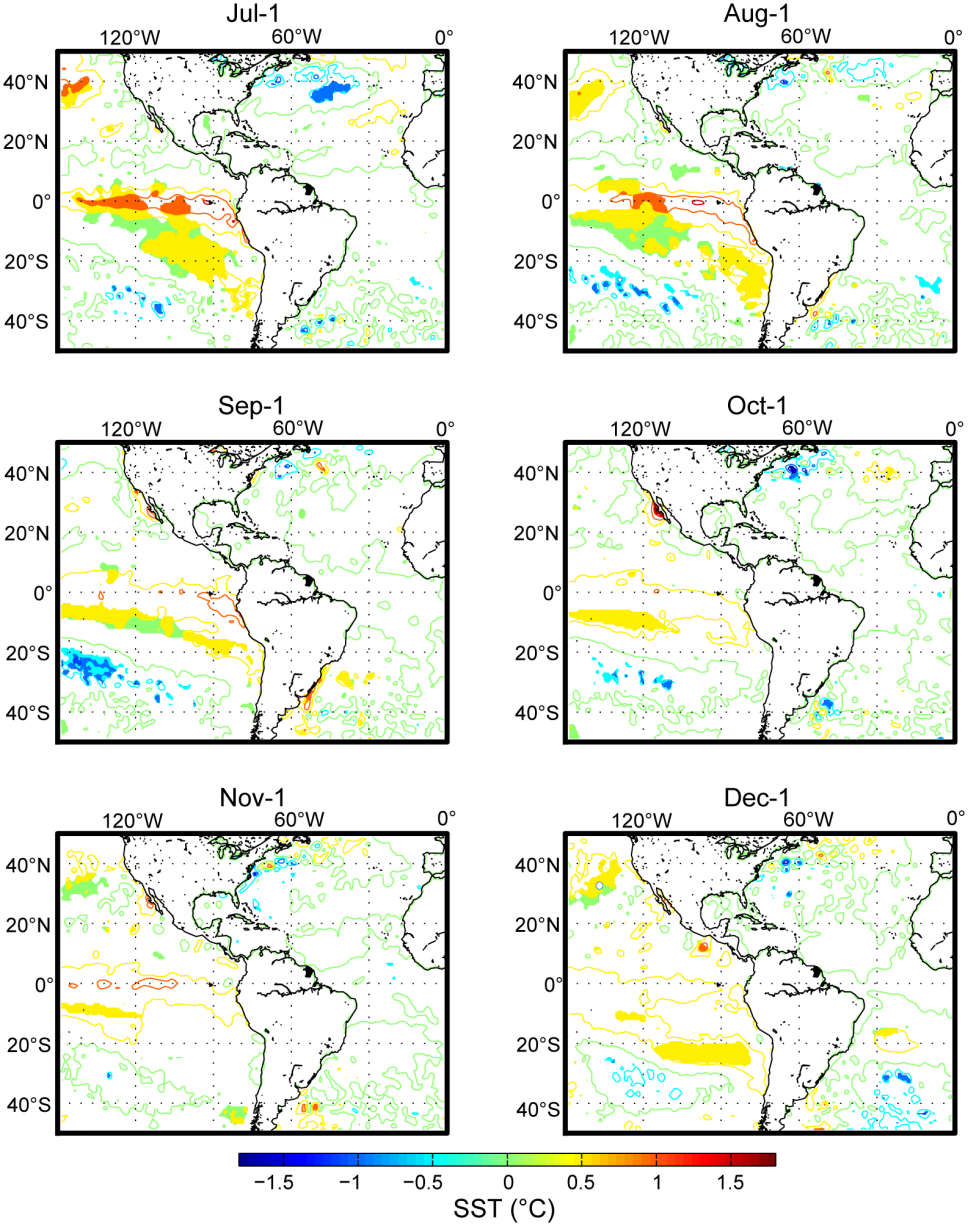
Sea surface temperature conditions that characterized an epidemic year. A composite analysis was performed by separately averaging the SST data for the years in which the highest (HIGH) and lowest (LOW) DF incidences were recorded in French Guiana. The contours (0.5°C interval) show the HIGH minus the LOW differences in the SST from July to December to illustrate the conditions that characterized a typical epidemic year. Filled-in areas indicate significant differences at the 5% confidence interval that were calculated using Student's *t*-test.

SST composite maps were calculated for the 12 months from January to December. The results indicated that epidemic years were characterized by increased Pacific Ocean SSTs during the pre-epidemic months of July and August (Fig 3, only July–December is shown here). This warming was particularly strong (approximately 1.5°C) at the equator at approximately 120°W, and the maximal spatial extent was observed in July.

**Fig 4:**
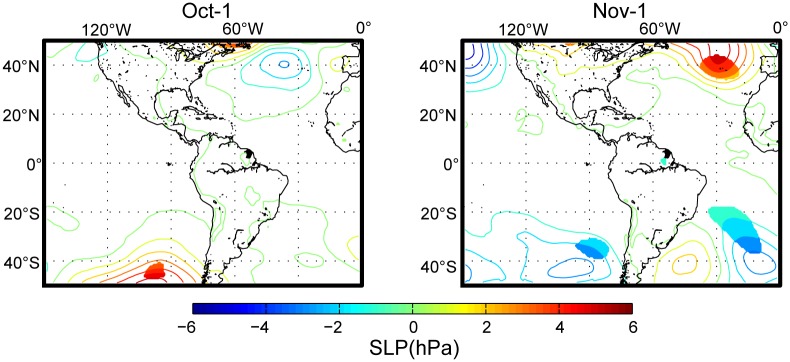
Sea-level pressure conditions that characterized an epidemic year. A composite analysis was performed by separately averaging the SLP data for the years in which the highest (HIGH) and lowest (LOW) DF incidence were recorded in French Guiana. The contours (at 0.5°C intervals) show the HIGH minus the LOW differences in the SLP from July to December to illustrate the conditions that characterized a typical epidemic year. Filled-in areas indicate significant differences at the 5% confidence interval and were calculated using Student's *t*-test.

The analysis of differences in atmospheric circulation between epidemic and non-epidemic years at the end of the dry season in October–November (when there were significant negative correlations between DF and rainfall; Fig 2) showed that epidemic years were characterized by northward positioning and a strengthening of the Azores High in November (Fig 4). The mean difference between epidemic and non-epidemic years was approximately 5 hPa and was maximal at 40°N, 30°W.

### Towards a predictive climate-based model

Based on previous results, the following climate indices were included in logistic binomial univariate and multivariate models (Table 1): (1) October–November, mean rainfall in French Guiana (FG-ON-RAIN); (2) July–August, mean Equatorial Pacific Ocean (2° N-20°S, 135°W-90°W) SST (EPO-JA-SST); and (3) November, Azores High (45°N-35°N, 40°W-20°W) SLP (AH-N-SLP). Because previous SST and SLP indices were found to be associated with rainfall in French Guiana, their common association in the same model was discarded.

**Table 1:** Model comparison of the logistic binomial regressions fitted to the DF outbreaks over the period 1991–2013.

	Univariate			Multivariate	
Covariates	FG-N-RAIN^a^	EPO-JA-SST^b^	AH-N-SLP^c^	EPO-JA-SST^b^	+ AH-N-SLP^c^
Unit	mm	°C	hPa	°C	hPa
**AIC**	30	30	31	27	
**AUC**	0.77	0.75	0.76	0.88	
**Coeff.**	-0.03	2.61	0.29	2.78	0.40
**Std. Err.**	0.02	1.42	0.15	1.33	0.20
**p-value**	0.048	0.073	0.061	0.036	0.045

AIC, Akaike information criterion; AUC, area under the curve; Coeff., coefficient; Std. Err., standard error.

^a^FG-N-RAIN, October–November mean rainfall;

^b^EPO-JA-SST, July–August mean Equatorial Pacific Ocean (2° N-20°S, 135°W-90°W) sea surface temperature;

^c^AH-N-SLP, November Azores High (45°N-35°N, 40°W-20°W) sea-level pressure.

**Fig 5:**
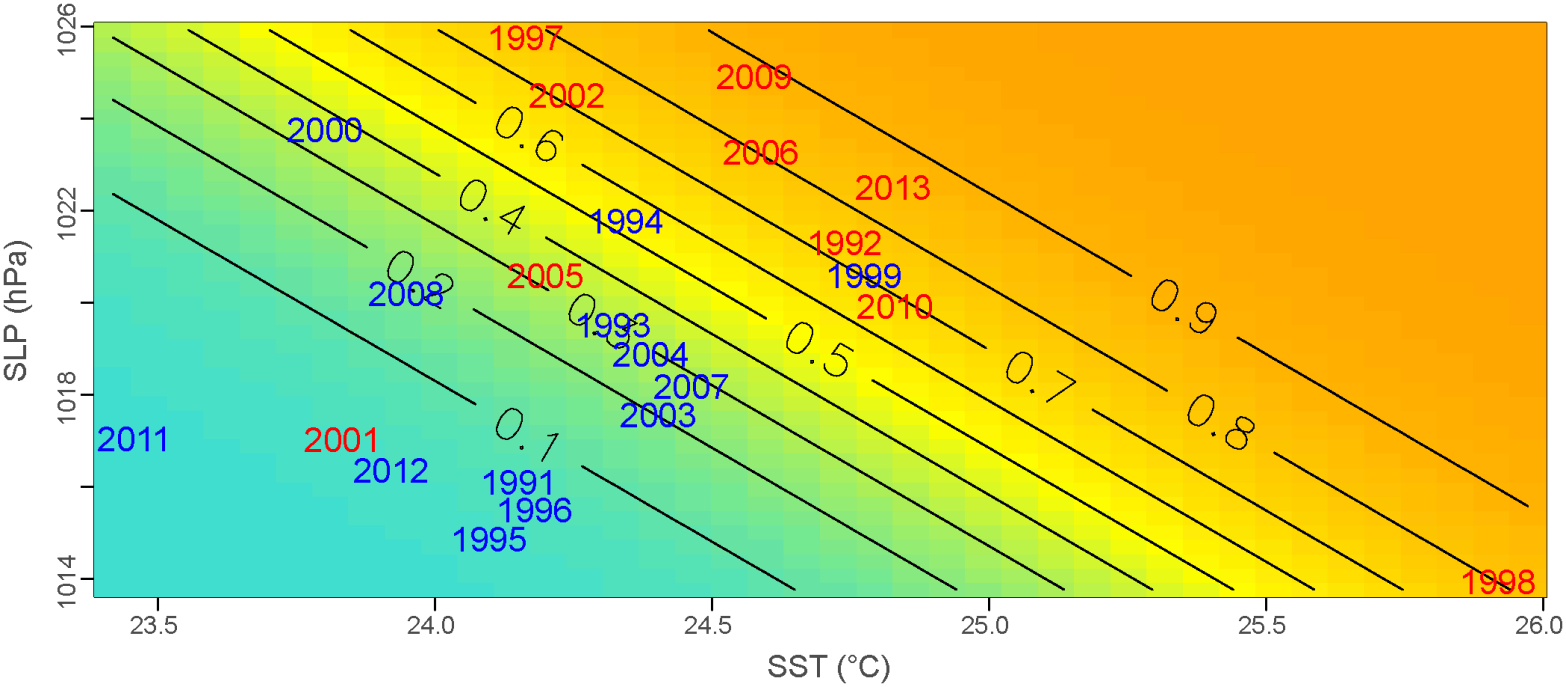
Logistic model probability and observed epidemiologic situations. The probability (grey lines) of an epidemic occurring in a year according to the July–August mean Equatorial Pacific Ocean (2° N-20°S, 135°W-90°W) SST and the November Azores High (45°N-35°N, 40°W-20°W) SLP values. In red (blue): epidemic (non-epidemic) years observed in French Guiana from 1991–2013.

**Fig 6:**
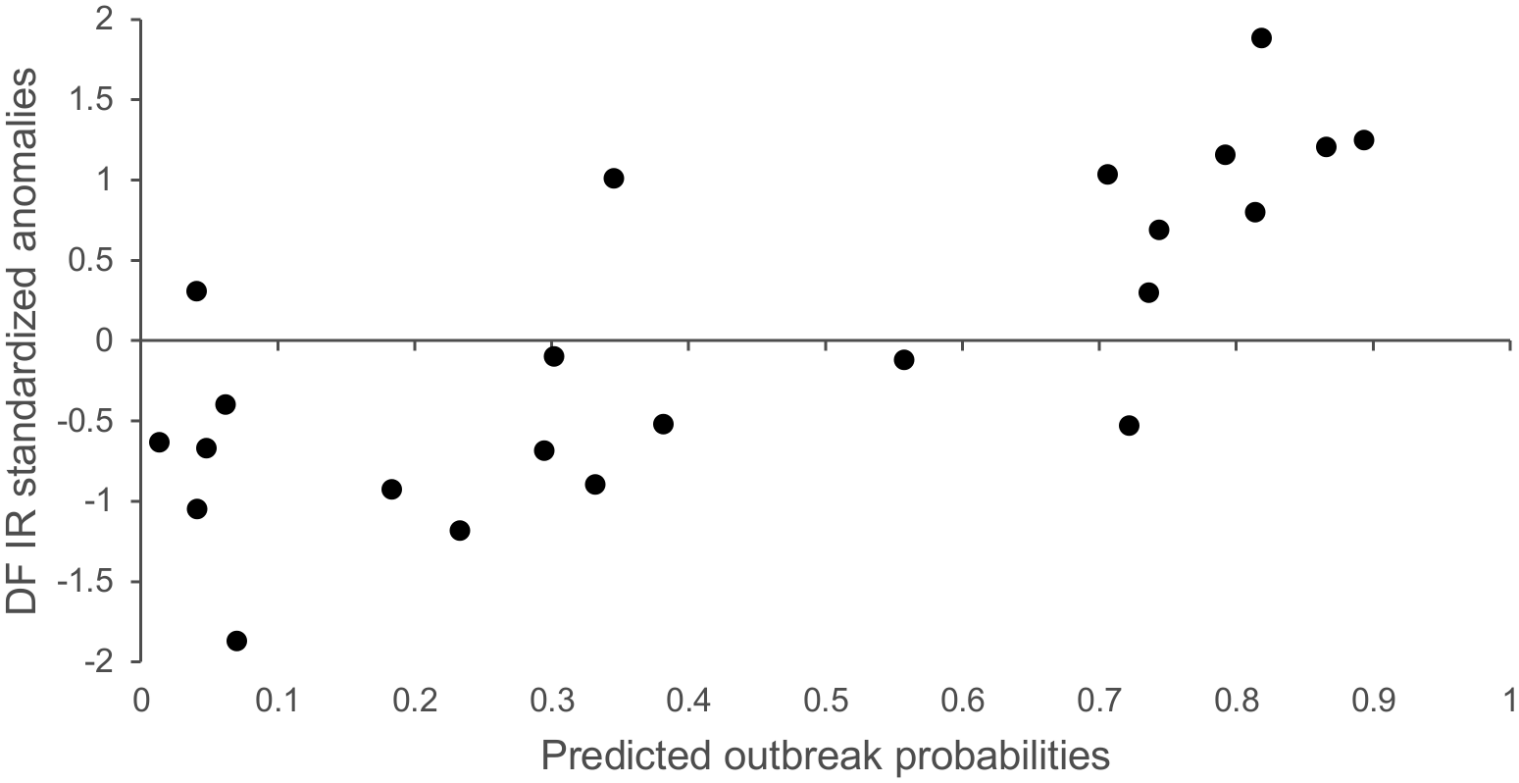
Relationship between the observed DF incidence rate standardized anomalies and predicted outbreak probabilities.

The multivariate model that included the two predictors EPO-JA-SST and AH-N-SLP yielded the best results for the AIC (27) and AUC (0.88), which suggested that it had good predictive value. Warming in the mean Equatorial Pacific Ocean SST in July–August and the strengthening of the Azores High in November greatly increased the probability that an outbreak would occur in the following year in French Guiana (Fig 5). Accordingly, 80% of the epidemic conditions were correctly predicted (HR = 0.80). Outbreaks in the years 2001 and 2005 were incorrectly predicted to be non-epidemic, and two years were predicted as false alarms (1994 and 1999). Finally, the LOOCV δ of 0.18 indicates that the model was robust and that only 18% of the years were misclassified when the LOOCV procedure was used. Yearly cross-validated probabilities are shown in S1 Fig. The scatter plot between the observed DF incidence rate standardized anomalies and the predicted outbreak probabilities (Fig 6) revealed a nearly linear relationship (Pearson’s correlation: r = 0.76; *P-value* < 0.01).

### What is the epidemic risk in 2016?

Forecasts for 2014 and 2015 (not included in the training dataset) indicated that the model predictions were consistent with the non-epidemic conditions that were observed in French Guiana (the DF IR/100 000 inhabitants was 350 in 2014 and 106 for January to September, 2015) (Table 2). In 2016, as a result of the warm SST conditions over the Equatorial Pacific Ocean that occurred in August and July (25.26°C) and the high pressures over the Azores High in November (1021.36 hPa), the model predicted that French Guiana would likely experience an outbreak (probability of 0.92).

**Table 2:** Outbreak predictions in French Guiana (2014–2016).

	2014	2015	2016 forecast
**AH-N-SLP (hPa)**^a^	1023.5	1018.69	1021.36
**EPO-JA-SST (°C)**^b^	23.98	24.45	25.26
**Observation**	NEPI	NEPI	?
**Forecast**	NEPI	NEPI	EPI
**Outbreak probability**	0.45	0.31	0.92

NEPI, non-epidemic year; EPI, epidemic year.

^a^AH-N-SLP, November Azores High (45°N-35°N, 40°W-20°W) sea-level pressure;

^b^EPO-JA-SST, July–August mean Equatorial Pacific Ocean (2° N-20°S, 135°W-90°W) sea surface temperature.

## Discussion

We investigated the relationship between climate and DF outbreaks in French Guiana to assess the possibility of including climate factors as predictors of epidemiological risk. Our findings highlighted a strong association between large-scale climate patterns and epidemic conditions in French Guiana. A simple and efficient statistical model was established to predict epidemic years. This model uses the summer Equatorial Pacific Ocean SST conditions six months prior to the mean onset month of the epidemic (July-1 –August-1) and the SLP of the Azores High three months prior (November-1), and it appropriately forecasted eight of the ten outbreaks that occurred in the 1991–2013 period. Outbreaks occurred after [i] warming in the Equatorial Pacific Ocean and [ii] northward displacement of the Azores High, which causes a rainfall deficit at the end of the dry season. This work refines the results of Gagnon et al. [37], who used the mean rainfall anomalies from May to April and concluded epidemics are associated with less abundant rainfall.

Warming events in the Equatorial Pacific Ocean are known to modify the mean climate over South America. During a warming phase, the northern region of the continent experiences drought conditions because the eastern subsidence of the Walker circulation is reinforced, which weakens convection and precipitation [48]. These results were consistent with the lagged correlation analysis, which indicated that a rainfall deficit would occur during the October and November preceding an epidemic year, and the *a priori* hypothesis derived from the work of Gagnon et al. [37], which indicated that there is an association between DF epidemic years in French Guiana and El Niño events. However, our analysis showed that anomalous SST patterns did not precisely correlate with El Niño events. Indeed, the SST anomalies of El Niño events were more intense in winter months. Importantly, certain epidemic years corresponded to strong (1997–1998) and moderate (1992, 2009–2010) El Niño events, although this association was not systematic. For example, an El Niño event did not occur from 2005–2006, but French Guiana did subsequently experience a dramatic epidemic.

In northern South America, rainfall is associated with water vapor that is transported from the north Atlantic via the northeasterly trade winds and thus from the SLP gradients. At the end of the dry season (October–November), the convergence of trade winds transports moisture over French Guiana, which fuels convection and precipitation. Positive SLP anomalies over the Azores High favor a northward position of the ITCZ over the Atlantic. Precipitation consequently decreases over the north part of South America in November.

Not all El Niño events are the same, and their effects on weather/climate may therefore differ. In 1994 and 1999, the model predicted an epidemic year would result from the high SLP and SST indices. However, the observed dry season rainfall patterns (rainfall surplus) were inconsistent with the usual occurrences, and this may explain the lack of an epidemic situation. In addition, 2001 and 2005 were epidemic years that were not predicted by the model. In these two years, dry season rainfall showed no positive or negative specific anomalies. For 2005, these observations were consistent (moderate SLP and SST indices versus moderate dry season rainfall). However, in 2001, given the low SST and SLP indices, a wetter dry season was expected than was observed. Thus, if a large part of the dry season rainfall variability in French Guiana is driven by the SST and SLP of the targeted areas, it is not always the case. These data highlight the complexity of predicting epidemiological patterns for non-climate-contrasted years and suggests that rainfall variability is not driven only by the two large scale indicators that were identified in the present study. Nevertheless, global predictions for the 1991–2013 period were better when the SST and SLP indices were used than when the dry season rainfall index was used (Table 1). Interestingly, the years that were incorrectly classified by the model preceded the implementation of the new enhanced surveillance system in 2006. Even when epidemic years that were identified by the tercile method were confirmed by historical reports, the representativeness of the surveillance system prior to 2006 could not differentiate an increased incidence that was caused by the presence of multiple isolated clusters from a major generalized epidemic. Historical reports may have overestimated the number of epidemiological circumstances, particularly in 2005, when one additional laboratory was included in the serological diagnosis process. Furthermore, three of the four misclassified years showed intermediate incidence rates (2^nd^ tercile) that corroborated our results. Specifically, the two identified false alarms (1994 and 1999) showed relatively high incidence rates for non-epidemic years. In addition, one of the two unpredicted epidemic years (2001) was associated with lower incidence rates than the other epidemic years.

Our findings indicate that an important rainfall deficit at the end of the dry season enhances the risk of epidemic in the following year, and these types of conditions are likely to impact the vector population. Two non-exclusive main hypotheses related to mosquito densities can be stated. First, the eggs of *Ae*. *aegypti*, which is the only urban vector for DF in French Guiana, are known to be able to resist desiccation and to thereby survive dry episodes [49]. During a particularly dry season, the majority of the breeding sites dry up, but when the first rains of the wet season occur, their breeding sites are once again hydrated, and their eggs hatch synchronously, resulting in a rough proliferation of adult mosquitoes that is favorable to the emergence of an epidemic via to their introduction to infectious patients. The second hypothesis is related to human behaviors. Although precipitation is known to contribute to the multiplication of breeding sites, drought can also indirectly expand the vector’s range. Indeed, during pronounced dry seasons, some people may adapt their lifestyles by maintaining additional water-collection containers. Thus, because of increases in breeding sites around and within households, *Ae*. *aegypti* can maintain significant background densities during the dry season. As a consequence, the virus can remain in the area during the dry season, leading to a higher potential of an epidemic when the wet season returns. Further entomological field investigations should be performed to test these hypotheses. The evolution of the surveillance system that was used for data collection beginning in 2006, following the introduction of new laboratories and new methods of diagnosis, increased the difficulty of performing meaningful comparisons of the scope of epidemics, particularly those that occurred before 2006. It is also important to take the circulating serotypes into consideration to enhance the assessment of the model’s predictions. Circulating serotypes that have affected only a small portion of the population before the predicted year could play a role by increasing the transmission risk, given the size of the susceptible population. Conversely, serotypes that have recently caused epidemics could limit the transmission risk despite propitious climatic conditions. In this study, we explored the reason that the model wrongly predicted certain epidemiological situations looking at the predominant serotypes. Two of the four years that were wrongly predicted could be explained by the serotypes circulating during the previous year. In 1999, for which the model erroneously predicted an epidemic, both circulating serotypes (DENV1 and DENV4) had already caused epidemics in 1997 and 1998. In addition, the 2001 epidemic, which was not predicted by the model, was caused by DENV3, which had not caused an epidemic in the ten previous years. Other well-known factors that might play a key role in transmission, including the immune status of the host population or the presence of outbreaks in neighboring countries, were not included in the present analysis. Finally, considering the potential competitive viral suppression in vectors that can be caused by co-infections, the emergence of new viruses that can also be transmitted by *Aedes* mosquitoes in French Guiana, such as the Zika virus, could limit the transmission of dengue fever. It will be interesting to see how the emergence of the Zika virus in 2016 may interacts with dengue fever transmission in a propitious climatic context.

Future studies should attempt to validate hypotheses regarding the impact of the identified climate factors and associated meteorological patterns (i.e., the rainfall deficit at the end of the dry season) on direct measurements of vector behavior and breeding sites. Among other possible future developments, we plan to take into account the sub-country incidence of dengue to model the propagation of epidemics within the country.

### Conclusions

Among the wide panel of factors that can influence DF outbreaks, these results suggest that large-scale climate factors play an important role. We found that the climatic indices that were assessed in this study were important for DF monitoring and for predicting outbreaks in French Guiana over a period of 2–3 months. This delay may give public health authorities the ability to anticipate outbreaks and implement social communication and vector control measures, and to adapt healthcare capacity and increase preparedness in a timely manner. Importantly, this model could be easily and regularly updated using newly collected data that was retrieved from the ongoing dengue surveillance system. Because the identified climate indicators are simple and easy to access, they could be used to estimate the probability of future epidemics occurring according to climate change simulations and help to evaluate the effectiveness of potential intervention strategies.

## Supporting Information

S1 Fig Predicted outbreak probabilities from the leave-one-out cross-correlation. The gray lines represent each individual forecast produced during the cross-validation process.
(TIFF)
