# Supplementary figures and images for: Predicting Dengue Fever Outbreaks in French Guiana Using Climate Indicators
Source: PLoS Negl Trop Dis. 2016 Apr 29;10(4):e0004681. doi: 10.1371/journal.pntd.0004681 (PMC4851397; doi:10.1371/journal.pntd.0004681)

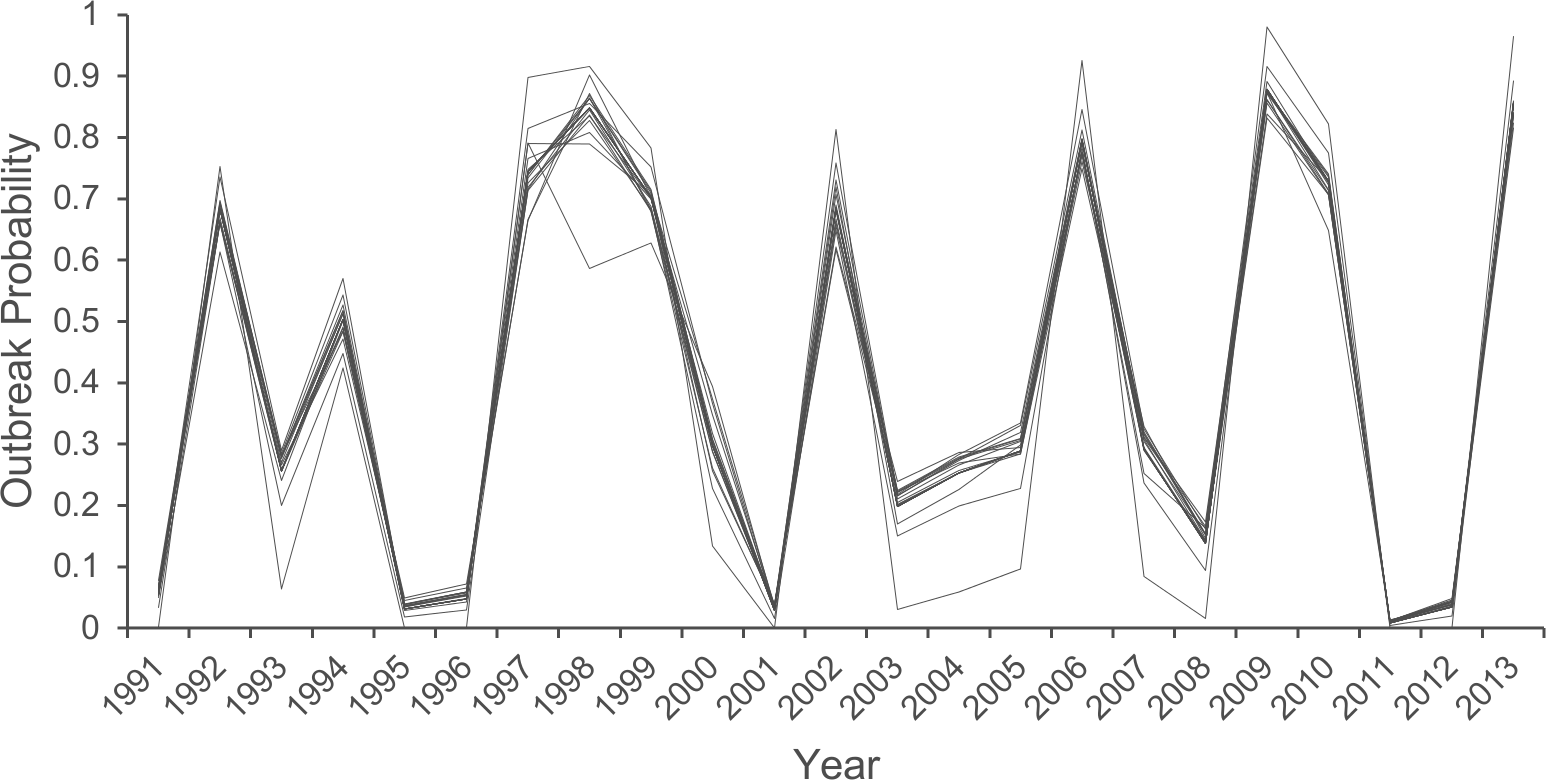

Supplement: S1 Fig — The gray lines represent each individual forecast produced during the cross-validation process. (TIFF) [file pntd.0004681.s001.tiff]
